# Supplementary material for: Hot spot prediction in protein-protein interactions by an ensemble system
Source: BMC Syst Biol. 2018 Dec 31;12(Suppl 9):132. doi: 10.1186/s12918-018-0665-8 (PMC6311905; doi:10.1186/s12918-018-0665-8)
Supplement: Supplementary file 2 — All relevant MATLAB code. A simple Matlab implement of our predictor. (ZIP 19,742 kb) [file 12918_2018_665_MOESM2_ESM.zip › Additional file 2/Read ASA/ASA website/BID/1CDLE.htm]

 NetSurfP - Protein Surface Accessibility and Secondary Structure Predictions

  

|  |  |  |
| --- | --- | --- |
|  |  | NetSurfP - Protein Surface Accessibility and Secondary Structure Predictions  Technical University of Denmark |

  


---

```
# For publication of results, please cite:
# A generic method for assignment of reliability scores applied to solvent accessibility predictions.
# Bent Petersen, Thomas Nordahl Petersen, Pernille Andersen, Morten Nielsen and Claus Lundegaard
# BMC Structural Biology 2009, 9:51 doi:10.1186/1472-6807-9-51
#
# Column 1: Class assignment - B for buried or E for Exposed - Threshold: 25% exposure, but not based on RSA
# Column 2: Amino acid
# Column 3: Sequence name
# Column 4: Amino acid number
# Column 5: Relative Surface Accessibility - RSA
# Column 6: Absolute Surface Accessibility
# Column 7: Z-fit score for RSA prediction
# Column 8: Probability for Alpha-Helix
# Column 9: Probability for Beta-strand
# Column 10: Probability for Coil
E A  1CDLE                  1    0.879  96.910  -0.018   0.016   0.005   0.979
E R  1CDLE                  2    0.640 146.652  -0.011   0.268   0.043   0.689
E R  1CDLE                  3    0.517 118.370   0.216   0.354   0.048   0.598
E K  1CDLE                  4    0.553 113.732   1.105   0.455   0.046   0.498
B W  1CDLE                  5    0.231  55.628  -0.884   0.455   0.046   0.498
E Q  1CDLE                  6    0.535  95.622   0.374   0.502   0.102   0.396
E K  1CDLE                  7    0.608 125.168   0.073   0.561   0.047   0.393
E T  1CDLE                  8    0.360  49.863  -0.559   0.455   0.046   0.498
E G  1CDLE                  9    0.394  30.976  -1.688   0.522   0.016   0.462
E H  1CDLE                 10    0.403  73.233  -0.256   0.622   0.015   0.363
B A  1CDLE                 11    0.210  23.153  -0.498   0.751   0.050   0.199
B V  1CDLE                 12    0.153  23.470  -0.257   0.779   0.100   0.120
E R  1CDLE                 13    0.429  98.310   0.359   0.779   0.100   0.120
B A  1CDLE                 14    0.275  30.327  -0.470   0.701   0.107   0.192
B I  1CDLE                 15    0.207  38.314   0.001   0.701   0.107   0.192
E G  1CDLE                 16    0.430  33.833  -1.232   0.605   0.105   0.290
E R  1CDLE                 17    0.551 126.248   0.160   0.386   0.097   0.517
E L  1CDLE                 18    0.382  70.017  -1.254   0.268   0.043   0.689
E S  1CDLE                 19    0.674  78.958  -1.103   0.181   0.016   0.803
E S  1CDLE                 20    0.919 107.660  -0.018   0.003   0.003   0.994

Explain the output.  Go back.
```

---
